# High Rates of Genome Rearrangements and Pathogenicity of *Shigella* spp.

**DOI:** 10.3389/fmicb.2021.628622

**Published:** 2021-04-12

**Authors:** Zaira Seferbekova, Alexey Zabelkin, Yulia Yakovleva, Robert Afasizhev, Natalia O. Dranenko, Nikita Alexeev, Mikhail S. Gelfand, Olga O. Bochkareva

**Affiliations:** ^1^Faculty of Bioengineering and Bioinformatics, Lomonosov Moscow State University, Moscow, Russia; ^2^Institute for Information Transmission Problems (The Kharkevich Institute, RAS), Moscow, Russia; ^3^Computer Technologies Laboratory, ITMO University, Saint Petersburg, Russia; ^4^JetBrains Research, Saint Petersburg, Russia; ^5^Bioinformatics Institute, Saint Petersburg, Russia; ^6^Department of Cytology and Histology, Saint Petersburg State University, Saint Petersburg, Russia; ^7^Skolkovo Institute of Science and Technology, Moscow, Russia; ^8^Institute of Science and Technology (IST Austria), Klosterneuburg, Austria

**Keywords:** *Shigella*, *Escherichia coli*, pathogens, recombination, E3 ubiquitin-ligases, IS, rearrangements

## Abstract

*Shigella* are pathogens originating within the *Escherichia* lineage but frequently classified as a separate genus. *Shigella* genomes contain numerous insertion sequences (ISs) that lead to pseudogenisation of affected genes and an increase of non-homologous recombination. Here, we study 414 genomes of *E. coli* and *Shigella* strains to assess the contribution of genomic rearrangements to *Shigella* evolution. We found that *Shigella* experienced exceptionally high rates of intragenomic rearrangements and had a decreased rate of homologous recombination compared to pathogenic and non-pathogenic *E. coli*. The high rearrangement rate resulted in independent disruption of syntenic regions and parallel rearrangements in different *Shigella* lineages. Specifically, we identified two types of chromosomally encoded E3 ubiquitin-protein ligases acquired independently by all *Shigella* strains that also showed a high level of sequence conservation in the promoter and further in the 5′-intergenic region. In the only available enteroinvasive *E. coli* (EIEC) strain, which is a pathogenic *E. coli* with a phenotype intermediate between *Shigella* and non-pathogenic *E. coli*, we found a rate of genome rearrangements comparable to those in other *E. coli* and no functional copies of the two *Shigella*-specific E3 ubiquitin ligases. These data indicate that the accumulation of ISs influenced many aspects of genome evolution and played an important role in the evolution of intracellular pathogens. Our research demonstrates the power of comparative genomics-based on synteny block composition and an important role of non-coding regions in the evolution of genomic islands.

## Importance

Pathogenic *Escherichia coli* strains frequently cause infections in humans. Many diverse *E. coli* strains exist in nature and their ability to cause disease is fueled, in particular, by incorporating novel genetic information via extensive horizontal gene transfer of plasmids and pathogenicity islands. The emergence of antibiotic-resistant *Shigella* spp., which are pathogenic forms of *E. coli*, coupled with the absence of an effective vaccine against them, highlights the importance of the continuing study of these pathogenic bacteria. Our study contributes to the understanding of genomic properties associated with molecular mechanisms underpinning the pathogenic nature of *Shigella*. We characterize the contribution of insertion sequences to the genome evolution of these intracellular pathogens and suggest a role of upstream regions of chromosomal *ipaH* genes in the *Shigella* pathogenesis. The methods of rearrangement analysis developed here are broadly applicable to the analysis of genotype-phenotype correlation in historically recently emerging bacterial pathogens.

## Introduction

*Escherichia coli* is likely the best-studied organism, at least on the molecular level. It is widely used to study fundamental aspects of bacterial genomics and is the subject of extensive research as an important component of the normal gut microbiota of vertebrates, including humans. While most *E. coli* strains are harmless, a non-negligible fraction is pathogenic, causing such diseases as diarrhoea, urinary tract infection, or meningitis (Zhang and Lin, 2012; The et al., 2016). Another group of pathogens, *Shigella*, which causes a severe form of bacillary dysentery, evolved from *E. coli* (Pupo et al., 2000; The et al., 2016). *Shigella* spp. are polyphyletic relative to *E. coli*, but the genus name is maintained in part due to the medical tradition (Pupo et al., 2000; Lan and Reeves, 2002; Chaudhuri and Henderson, 2012). Nevertheless, from an evolutionary perspective, *Shigella* is just a set of strains causing a specific disease within the broader *E. coli* phylogenetic group.

*Shigella* strains carry a large plasmid (*pINV*) which is essential for virulence (Pupo et al., 2000; Lan and Reeves, 2002; Lan et al., 2004; Yang et al., 2007; Beld and Reubsaet, 2012; The et al., 2016). They also might be distinguished from *E. coli* by their non-motility with the associated absence of decarboxylated lysine, and by various biochemical characteristics, such as inability to ferment lactose and mucate (Pupo et al., 2000). One more *E. coli* pathovar, enteroinvasive *E. coli* (EIEC), generally exhibits the same pathogenic and biochemical features as *Shigella*, including invasiveness provided by *pINV* (Pupo et al., 2000; Lan and Reeves, 2002; Lan et al., 2004; Yang et al., 2007; Beld and Reubsaet, 2012; The et al., 2016). Such phenotypic similarity may be attributed to adaptation to similar environmental conditions as *Shigella* and EIEC spend most of their lifecycle inside eukaryotic cells, while most *E. coli* strains inhabit extracellular space. Thus, EIEC could represent either a *Shigella* “prototype,” which could be a precursor for a typical *Shigella*, or a distinct group of pathogenic *E. coli* that have adapted to an intracellular lifestyle but, unlike *Shigella*, have not lost the ability to live outside eukaryotic cells (Lan and Reeves, 2002; Lan et al., 2004; Beld and Reubsaet, 2012).

Acquisition of the virulence plasmid enabling intracellular lifestyle was likely a key event of the *Shigella* evolution that facilitated further adaptation. This evolution may have involved a variety of events, such as point mutations, suppression of certain genes, deletion of anti-virulence genes, or acquisition of insertion sequences (ISs). On the other hand, the intracellular niche may have provided a more relaxed selective pressure due to abundant resources and lack of competitors (Beld and Reubsaet, 2012; The et al., 2016) that, in conjunction with lower effective population size, would have decreased the negative selection rate (Mamirova et al., 2007) and caused substantial changes in the genome arrangement and composition. *Shigella* genomes feature loss or inactivation of many genes, which has been attributed to the relaxation of selection acting on those genes (The et al., 2016). Gene deletions are believed to contribute to the specialisation of bacteria and to enable rapid adaptation to different conditions in the host cell (Achaz et al., 2003; Yang et al., 2005; Beld and Reubsaet, 2012).

The chromosome and plasmids of *Shigella* species contain many ISs, small mobile DNA fragments that easily translocate within the genome. An analysis of draft *Shigella* genomes demonstrated convergent loss of metabolic pathways by the integration of diverse ISs and pseudogenisation by point mutations, often leading to degradation of multiple genes in the same pathway (Hawkey et al., 2020). However, the impact of IS elements on genome rearrangements and chromosome evolution has not been studied due to limitations arising from the use of incompletely assembled genome drafts. Indeed, the same repeated IS elements that increase the chromosome instability yield difficulties for the genomes assembly (Shapiro and von Sternberg, 2005; Avershina and Rudi, 2015).

In turn, repeats accumulation leads to genome rearrangements and changes in the expression of adjacent genes (Buchrieser et al., 2000; Yang et al., 2005; Couturier and Rocha, 2006; Darling et al., 2008; Beld and Reubsaet, 2012; Siguier et al., 2014) affecting the bacterial phenotype. The type of rearrangement depends on the mutual arrangement of the repetitive elements that have been involved in the recombination event. Recombination between inverted repeats leads to inversions, recombination between direct repeats leads to deletions, and recombination between direct repeats during replication leads to duplication (Darling et al., 2008). Since large deletions, insertions, and duplications are often under negative selection and are rare, inversions are the main drivers of structural changes in bacterial chromosomes (Achaz et al., 2003). The frequency of rearrangements varies and may correlate with the presence of mobile genetic elements (Achaz et al., 2003; Darling et al., 2008).

Here, we provide a comprehensive analysis of the complete genomes of *E. coli* and *Shigella* strains, based on the construction of synteny blocks, and assess the contribution of ISs to the genome evolution of *Shigella* spp. We show that *Shigella* genomes have experienced exceptionally high rates of intragenomic rearrangements and a decreased rate of homologous recombination in comparison to other *E. coli* strains, including pathogenic ones. Then, we focus on the rates of expansion of different ISs families and the patterns of their integration in the genomes. Finally, we describe genome rearrangements that have occurred independently in separate lineages, showing the convergent evolution of *Shigella*.

## Materials and Methods

### Genomes

We used all complete and annotated genomes of *Shigella* and *E. coli* available in GenBank as of April 2019 (NCBI Resource Coordinators, 2018). We constructed a phylogenetic tree for *E. coli* strains only and excluded clones and closely related *E. coli* strains from further analysis to reduce the size of datasets with the minimal loss of diversity (Supplementary Figure 1a). Thus, for *E. coli* strains with identical names and on short branches, we selected a random one and removed all others. In particular, we used only one reference genome for *E. coli* K12, *E. coli* O157:H7, *E. coli* O104:H4, *E. coli* O145:H28, *E. coli* BH100, *E. coli* EcoI, *E. coli* O127:H6, *E. coli* O25b:H4, *E. coli* O55:H7, *E. coli* ST540, *E. coli* ST2747, *E. coli* BL21/DE3, *E. coli* Nissle 1917, *E. coli* clone D i2, *E. coli* MRSN, and *E. coli* AR strains (the list of excluded genomes is available on GitHub: https://github.com/zseferbekova/Shigella Project/tree/master/1Tree/Data/excluded_ strains.csv). In total, we analysed 414 complete genomes, including 35 *Shigella* spp., 41 STEC, 31 ExPEC, 8 APEC, 7 ETEC, 3 EPEC, 3 AIEC, 2 EAEC, and 1 EIEC genome (Supplementary Table 1).

### Phylogenetic Tree

**FIGURE 1:**
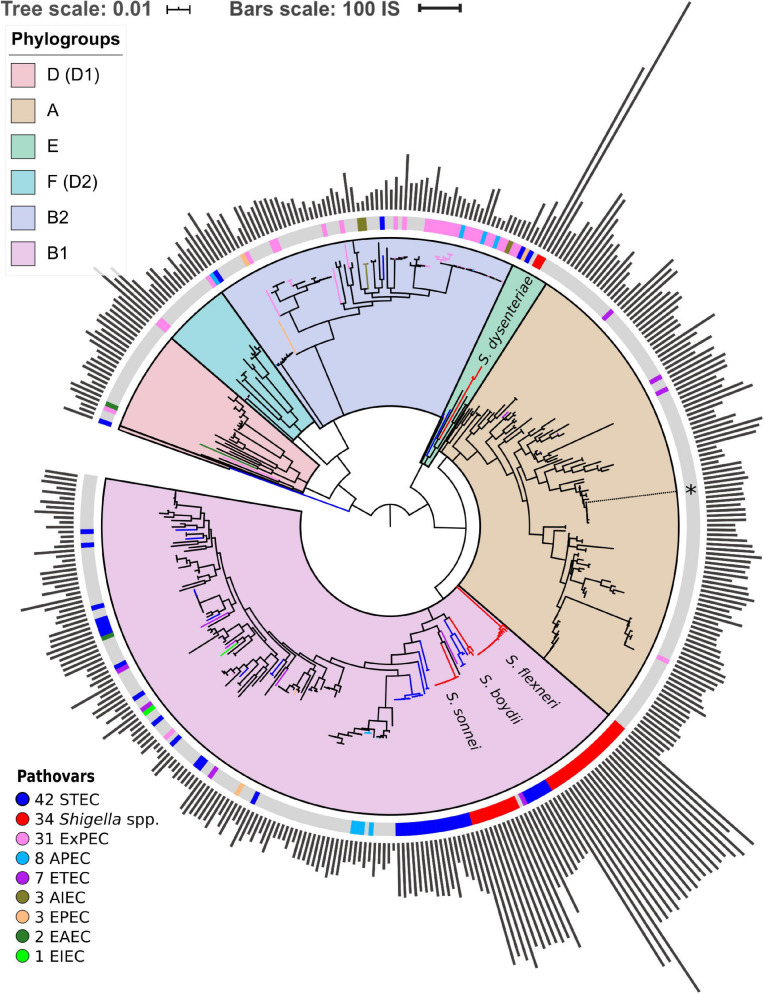
Phylogenetic tree of *Escherichia coli* and *Shigella* spp. The tree is based on the nucleotide alignment of universal single-copy orthologs. Clusters and corresponding phylogroups are shown in different colours; the number of genomes of each pathovar is indicated to the right of the pathovar’s name. Pathogenic *E. coli* strains are marked with coloured bands on the outer circle. The location of the unclassified *Shigella* strain is shown with an asterisk. The bars indicate the number of ISs found by ISsaga in bacterial chromosomes. The tree is unrooted.

For the construction of the strains’ phylogenetic tree, we used 238 universal, single-copy orthologous groups found in all 414 genomes. Orthologous groups were constructed using ProteinOrtho V5.13 (Lechner et al., 2011) with parameters cov = 67 (at least 67% coverage of both proteins in the BLAST alignment) and identity = 50 (at least 50% identity in the common segments). Then we constructed a nucleotide multiple sequence alignment of genes in each orthologous group using Mafft (Katoh and Standley, 2013) in the linsi mode. We then used RAxML (Stamatakis, 2014) with the GTR + Gamma model and 100 bootstrap replicates to construct a phylogenetic tree based on the concatenated alignment of these genes (Figure 1). Finally, we used the GGRaSP R package to divide all strains in the tree into seven clusters corresponding to the standard phylogroups (Clarke et al., 2018). All trees were visualised using online iTOL (Letunic and Bork, 2016).

Additionally, we checked the robustness of the phylogenetic tree with regards to the identity threshold in the definition of orthologs. For that, we reclustered the orthologs with the protein identity threshold = 95% and compared the phylogenetic trees (Figure 1 and Supplementary Figure 1b). The trees are consistent except for a small number of internal nodes with low bootstrap support. As we do not directly use the fine topology of the phylogenetic tree, that does not affect the results, nor does that influence the chromosome rearrangement analysis, as the latter has been performed directly on nucleotide sequences of the genomes.

### Insertion Sequence Elements

Online tool ISsaga (Varani et al., 2011) was used for annotation of IS elements in the chromosome sequences. For general statistical calculations, we used the number of predicted open reading frames (ORFs) associated with ISs. For more precise annotation of ISs from different families, we used the number of predicted ISs that could include several ORFs.

### Synteny Blocks

Multiple whole-genome alignment for the construction of locally-collinear blocks was performed with SibeliaZ (Minkin and Medvedev, 2020). As this approach had been specifically designed to address the performance issues caused by a large number of assembled genomes, it allowed us to scale our analysis to more than 400 genomes while maintaining accuracy. The *k*-mer size (−*k*) was set to 15, which is recommended by the documentation for bacterial genomes. Next, submodule maf2synteny from Ragout (Kolmogorov et al., 2018) was used to merge locally collinear blocks into synteny blocks (Supplementary Table 2). This approach is not sensitive to the annotation of genomes and identification of orthologs, since it is based on compacted de Bruijn graphs constructed directly for nucleotide genomic sequences. The minimal block size (−*b*) parameter was set to 1,000, the simplification parameter (−*s*) was set to fine in order to retain the information about small-scale rearrangements. These stringent parameters allowed us to extend the analysis of rearrangements to recent pseudogenes, RNA genes, and conserved intergenic regions. The location of the synteny blocks in the chromosomes was visualised using Circos (Krzywinski et al., 2009).

To infer the number of inversions on the phylogenetic tree we used common single-copy synteny blocks with the block size threshold (−*b*) of 5 kb. In each phylogenetic cluster, we used distance matrices where each element is a number of synteny blocks between given strains. We constructed trees based on the obtained matrices using PHYLIP (Feisenstein, 1989) and the neighbour-joining algorithm.

### Inversions Scenario

We reconstructed the history of inversion events using MGRA (Multiple Genome Rearrangements and Ancestors) software (Avdeyev et al., 2016). This tool takes as an input a phylogenetic tree and genomes represented as sets of synteny blocks. This analysis included chromosomes of all *Shigella*, related *E. coli* and several representative *E. coli* from each cluster not containing *Shigella* (51 genomes in total).

### Parallel Rearrangements

We say that a rearrangement is *consistent* with a tree if we may associate it with a particular branch on a tree, otherwise, we call a rearrangement *parallel*. We test each rearrangement for consistency with a tree with the standard Fitch algorithm (Erdõs and Székely, 1994). This approach allows us to detect the events occurring multiple times in distant clades, in particular, in different *Shigella* lineages.

To analyse inversions, we considered common single-copy blocks. For these 377 blocks, we constructed the breakpoint graph (Bafna and Pevzner, 1993; Alekseyev and Pevzner, 2009) as follows. The graph is built on 377 × 2 vertices. For each block *B*, we introduce two vertices *B*_H_ and *B*_T_, its head and tail, respectively. If two blocks *B* and *C* are adjacent in genome *g*, the vertices *B*_H_ and *C*_T_ are linked by an edge of colour *g*. We note that since we consider only common blocks and all genomes are circular, the edges of each colour form a perfect matching on the graph vertices. Since some adjacency edges of different colours may link the same pair of vertices, we introduce multi-edges – a multi-edge is a set of parallel edges of different colours. The breakpoint graph for our data contains 754 vertices and 656 multi-edges. If two strains differ from each other by one inversion, this corresponds to a four-cycle in the breakpoint graph (Supplementary Figures 2a,b). For each multi-edge, we split the set of strains into patterns depending on the presence of the corresponding colour edge in the multi-edge (Supplementary Figure 2c). Thus we associated each breakpoint (and each inversion) with a partition of the set of strains into patterns.

To analyse insertions, deletions, and duplications, we considered all blocks which were present in different copy numbers in some strains. For each block, we split the set of strains into patterns so that the copy number of this block in each pattern was the same (i.e., the pattern with strains containing zero copies of the block, the pattern with strains containing one copy of the block, etc). Thus, we associated each copy number variation with a partition of the set of strains into patterns. Then, for blocks whose copy numbers differ in *Shigella* and *E. coli*, we manually classified the evolutionary scenarios based on the occurrence pattern and functional annotation of genes found in the block.

The developed pipeline for the detection of parallel rearrangements is available on GitHub: https://github.com/ctlab/parallel-rearrangements.

### Rates of Homologous Recombination

For this analysis, we considered genomes from phylogroup B1 as it contains most of the *Shigella* strains, and for better resolution used pairwise full-genome alignments constructed using MAUVE (Darling et al., 2004). Then for fragments without gaps, we calculated the number of non-identical columns in each 1 kb segment. Thus, for each pair of genomes, we constructed the distributions of the number of mutations across the genomes.

In the case of strictly vertical inheritance, this distribution would be Poisson with the parameter λ reflecting the time of strain divergence. Fragments transferred horizontally from distant strains would contain more mutations yielding deviation from the Poisson distribution in the form of a heavy tail (Dixit et al., 2015). The latter, being a mixture of the Poisson distributions with unknown parameters may be fitted by the Erlang distribution.

We have used the Python SciPy module (Virtanen et al., 2020) to fit all pairwise distributions by the function *F*_λ,*k*,μ,*W*_(*X*)=**W**×**P**_λ_(*X*)+(1−*W*)×**E**_*k*,μ_(*X*), where **P**_λ_(*X*)=*e*^−λ^*λ*^*X*^/*X*! is the Poisson distribution with parameter λ, **E**_*k*,μ_(*X*)=(*X*/μ)^*k*−1^*e*^−*X*/μ^(μ(*K*−1)!) is the Erlang distribution with the shape *k* and scale μ [mode = (*k*–1)μ, mean = *k*μ, variance = *k*μ^2^], and the weight *W* in the range [0,1] measures the vertically inherited fraction of genome while (1–*W*) corresponds to the horizontally transferred fraction.

This approach extends the one suggested in Dixit et al. (2015). It averages over all genome segments, and hence is more robust than the approaches based on explicit identification of recombined segments, as the latter are sensitive to uneven evolutionary rates and, moreover, are computationally prohibitive for large-scale analyses. The Poisson parameter λ monotonically increases with the divergence time of the vertically inherited genome fraction, and selecting pairs with the same λ, we obtain a set of strain pairs that have diverged at approximately the same time.

## Results

### Structure of the Phylogenetic Tree and Accumulation of IS Elements

We found 238 universal single-copy orthologous groups in 414 genomes (Supplementary Table 1) and used them to construct the unrooted phylogenetic tree (Figure 1). The structure of the obtained phylogenetic tree recapitulates known *E. coli* phylogroups and supports the hypothesis that *Shigella* spp. included in our analysis evolved several times independently from *E. coli* and are named in accordance with the tree branches (Chaudhuri and Henderson, 2012; Dusek et al., 2018). One of the *Shigella* genomes (GenBankID: GCA_001596115.1) was unclassified and did not cluster with any described *Shigella* species. Moreover, the source of the sample was lichen, which is highly unusual and unlikely for *Shigella*. Thus, we assumed that in this case the taxonomic annotation was wrong and did not consider this genome as *Shigella*. The only complete and annotated EIEC strain did not cluster with any *Shigella* (Bordenstein and Reznikoff, 2005). Other pathogenic *E. coli* strains also did not form any monophyletic clusters.

**FIGURE 2:**
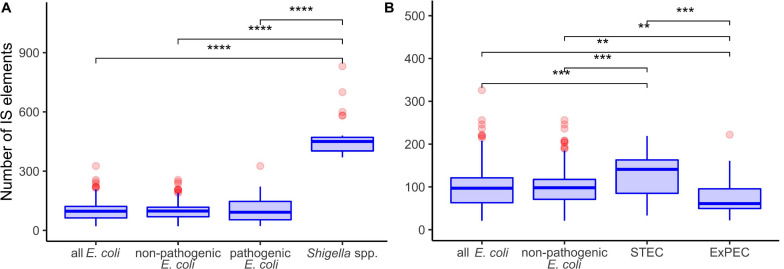
Comparison of insertion sequence (IS) numbers in panel **(A)** *Shigella* spp., non-pathogenic *E. coli*, other pathogenic *E. coli*, and all *E. coli* (excluding *Shigella* spp.); **(B)** two most abundant pathovars – ExPEC and STEC, non-pathogenic *E. coli*, and all *E. coli* (excluding *Shigella* spp.). The Wilcoxon–Mann–Whitney test with the Bonferroni correction: **p ≤ 0.01; ***p ≤ 0.001; ****p ≤ 0.0001.

*Shigella* genomes generally encode more IS elements than non-pathogenic *E. coli* (Yang et al., 2005). To estimate the density of IS elements, we calculated their number in the chromosomes of all 414 strains (Figure 1). The number of IS elements in *Shigella* genomes was significantly higher than in the genomes of pathogenic and non-pathogenic *E. coli* strains (Figure 2A; the Wilcoxon–Mann–Whitney test, *p*-value = 2.4 × 10^–21^ and 7.8 × 10^–18^, respectively). Interestingly, the two most sequenced pathovars, STEC and ExPEC, had, respectively, significantly higher (*p* = 1.9 × 10^–4^) and lower (*p* = 3.5 × 10^–4^) number of IS elements than the average in non-pathogenic *E. coli* (Figure 2B).

**FIGURE 3:**
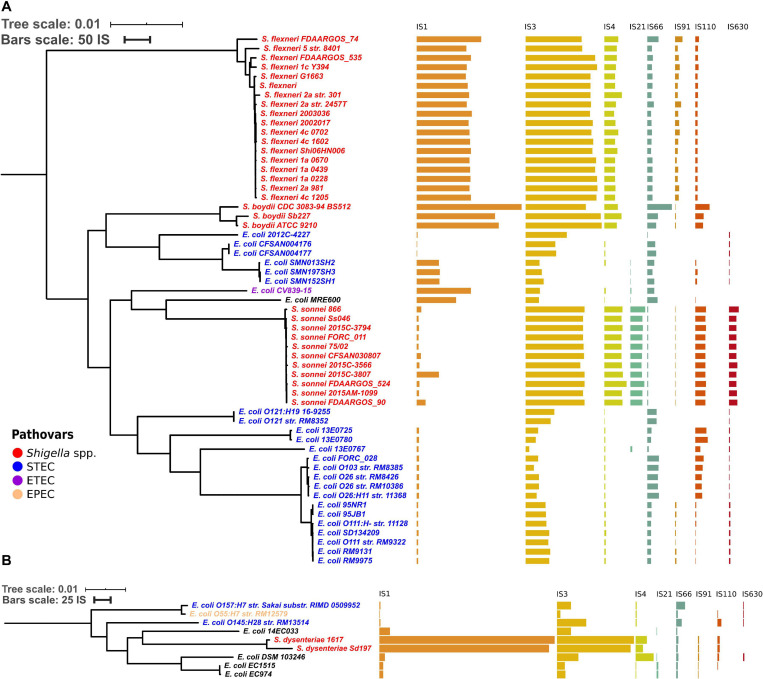
Distribution of IS families in panel **(A)** a part of phylogroup B1 and **(B)** phylogroup E. The bars indicate the number of ISs in each family.

**FIGURE 4:**
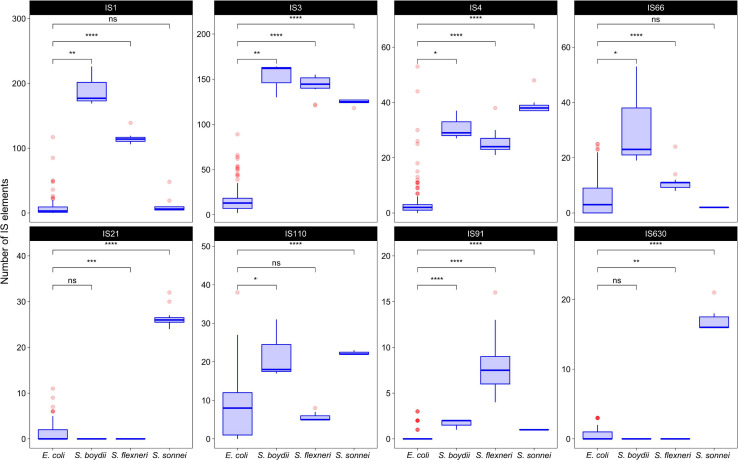
The comparison of selected IS families in *Shigella* and *E. coli* strains from phylogroup B1. The Wilcoxon–Mann–Whitney test with the Bonferroni correction: *p ≤ 0.05; **p ≤ 0.01; ***p ≤ 0.001; ****p ≤ 0.0001, ns, non-significant.

To test whether the distribution of IS families differed in two clusters of *Shigella* we merged IS elements into larger families and mapped the results on the phylogenetic tree (Figures 3, 4). We found four IS families (IS1, IS3, IS4, and IS91) which were enriched in all *Shigella* in comparison to *E. coli*. Moreover, we detected IS families that were specific for some *Shigella* lineages (IS21, IS110, IS630, and IS66). However, as our dataset included only three *Shigella boydii* and two *Shigella dysenteriae* genomes, the results for these lineages should be considered preliminary. By contrast, the *Shigella sonnei* group was represented by 11 strains and showed a significantly higher number of IS21, IS110, and IS630 elements (the Wilcoxon–Mann–Whitney test, *p* = 1.3 × 10^–8^, 5.1 × 10^–7^, 1.9 × 10^–9^, respectively).

### Composition of Synteny Blocks

**FIGURE 5:**
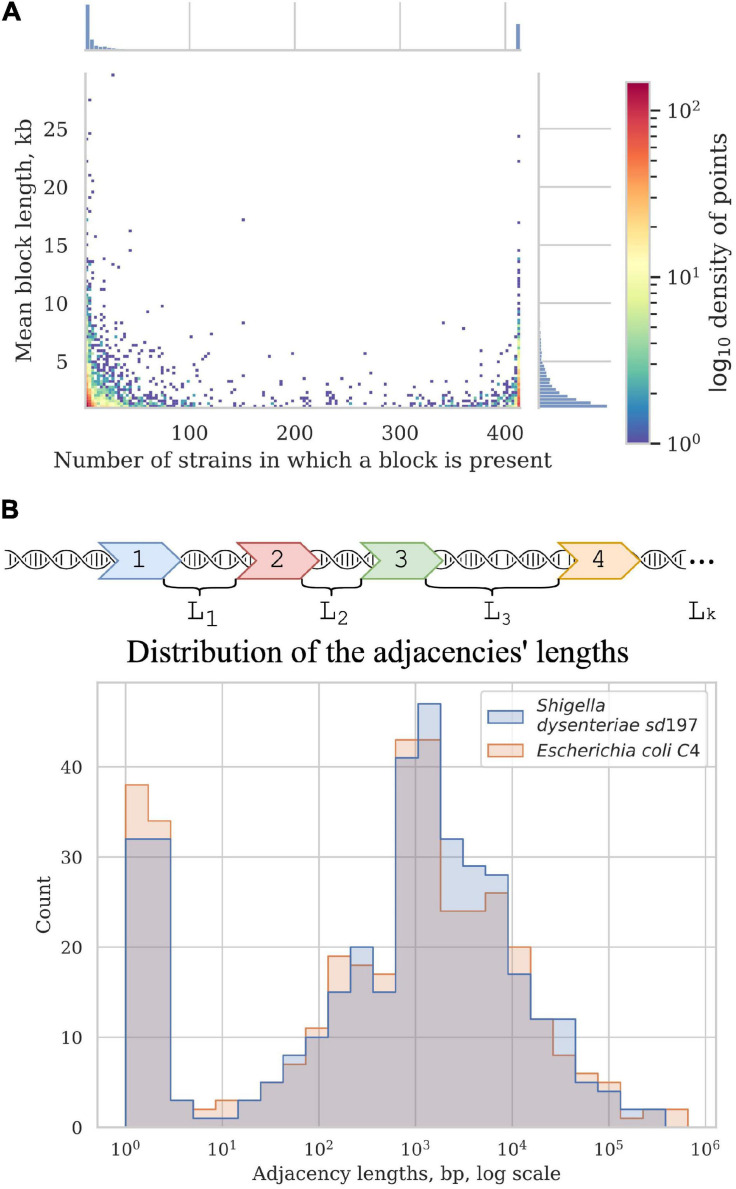
Synteny blocks. **(A)** 2D histogram shows the relationship between the mean block length and the number of strains in which it is present. The colour of a point corresponds to the number of synteny blocks with these parameters. *X*-axis histogram shows the frequency distribution of the synteny blocks; *Y*-axis histogram shows the length distribution of the synteny blocks. **(B)** Distribution of the adjacencies’ lengths in *E. coli* and *Shigella*, in nucleotides. Adjacencies are defined as the chromosome regions between the neighbouring universal synteny blocks (see section “Materials and Methods”).

In all studied *Shigella* and *E. coli* genomes we found 4,019 synteny blocks. The distribution of the synteny blocks by the number of strains in which they are present has an asymmetric *U*-shaped form similar to the distribution of gene frequencies in a population, also known as the *U-curve* (Figure 5A). Only 377 synteny blocks were classified as universal, so that each block was found exactly once in all considered genomes, while other 3,642 synteny blocks were not found, or found more than once, in at least one genome. The mean fraction of a chromosome covered by synteny blocks with the length threshold of 1 kb was 62%. The universal blocks spanned only 25–29% of the chromosome length and the distribution of these blocks across the chromosomes was not uniform, with long sections not harbouring any universal blocks (Figure 5B). The comparison of the distributions of common blocks across the chromosomes in different *Shigella* lineages, combined with GC-skew plots, revealed numerous unbalanced genomic rearrangements.

Most blocks are shorter than 5 kb; some exceptionally long blocks are found among both common blocks and blocks that are present in only a few strains (Figure 5A). Common long blocks are formed by operons of housekeeping genes, the longest ones being: 20 genes including DNA polymerase III subunit alpha, components of the complex involved in the assembly of outer membrane proteins and elongation factors; 19 genes including components of the cell division complex and the *mur* operon; and 14 genes that encode components of the NADH-ubiquinone oxidoreductase complex. The longest rare blocks are formed by recent insertions such as prophages and pathogenicity islands.

The focus on the presence/absence patterns of synteny blocks allowed us to distinguish between genome rearrangements not affecting copy numbers (such as inversions) and those leading to copy number variations (such as insertions, deletions, and duplications). We then separately constructed the breakpoint graph for universal blocks and analysed phyletic patterns of non-universal blocks (see section “Materials and Methods”). Thus we identified genetic features that could not be parsimoniously explained by common ancestry.

### Rates of Genome Rearrangements

*Shigella* genomes are thought to be dynamic due to numerous IS elements that promote non-homologous recombination (Buchrieser et al., 2000; Beld and Reubsaet, 2012; Siguier et al., 2014). Thus, the rate of rearrangement in *Shigella* genomes may be higher in proportion to the accumulation of single nucleotide substitutions in comparison to non-invasive pathogenic *E. coli*. To test this possibility, we matched the number of syntenic blocks with the number of single nucleotide substitutions in the universal genes for pairs of genomes in each phylogroup. The number of single-copy synteny blocks common to two genomes was inversely proportional to the collinearity of the genomes, i.e., more collinear genomes had fewer blocks while more blocks corresponded to genomes with a large number of inversions. While the number of synteny blocks may not accurately reflect the number of rearrangement events, especially when the number of such events is substantial, our approach provides a lower bound estimate of the number of inversion events. We then plotted the number of synteny blocks relative to the number of single nucleotide substitutions for each pair of genomes (Supplementary Figures 3, 4).

**FIGURE 6:**
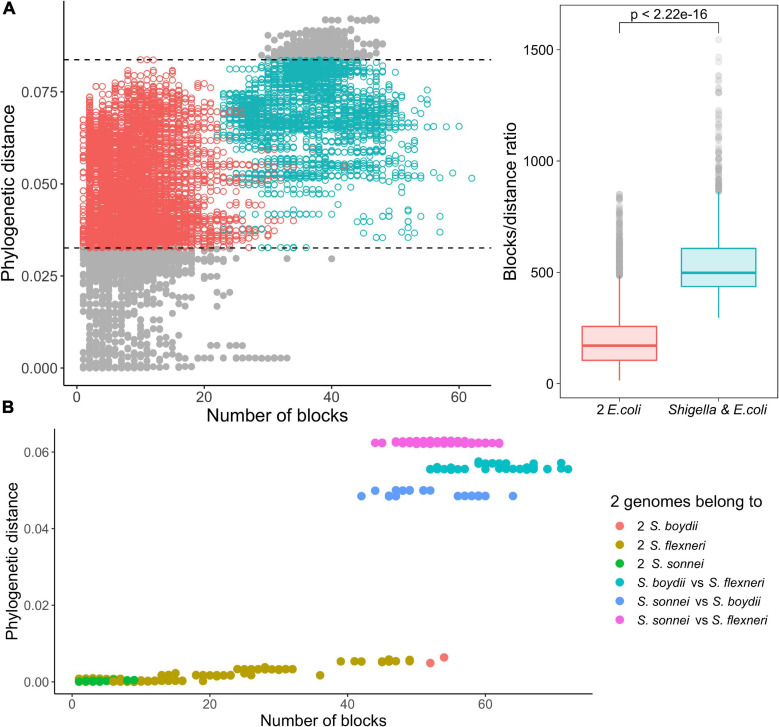
Comparison of the ratio of phylogenetic distance to the number of synteny blocks for genome pairs from phylogroup B1. **(A)** Each pair consists of one *Shigella* spp. and one *E. coli* genome (the red dots) or two *E. coli* genomes (the green dots). Only genome pairs in the intersection were compared. The Wilcoxon–Mann–Whitney test *p*-value is given. **(B)** Each pair consists of two *Shigella* genomes.

Indeed, within the same interval of evolutionary distances between strains, in pairs of *Shigella* and *E. coli* strains the ratio of a number of synteny blocks to single nucleotide substitutions was substantially higher, in comparison to pairs of *E. coli* strains (the Wilcoxon–Mann–Whitney test, *p* = 2.22 × 10^–16^) (Figure 6A). Moreover, for pairs of *Shigella* this ratio is even higher and different in four *Shigella* lineages (Figure 6B). Thus, genome rearrangements were occurring more frequently in *Shigella* history compared to *E. coli*. In other pathogenic *E. coli*, the ratio of a number of synteny blocks to single nucleotide substitutions did not differ from the average in non-pathogenic strains (Supplementary Figures 4a–d). The unclassified *Shigella* strain from lichen did not differ from *E. coli* strains (Supplementary Figure 4a), further supporting our assumption that this strain has been misclassified.

### Parallel Genome Rearrangements

Events that occur multiple times independently on a phylogeny, called homoplasies, could indicate selection pressure acting on populations adapting to an intracellular lifestyle. Here we focus on events that have occurred several times independently in the *Shigella* lineages.

#### Inversions and Rearrangement Hotspots

**FIGURE 7:**
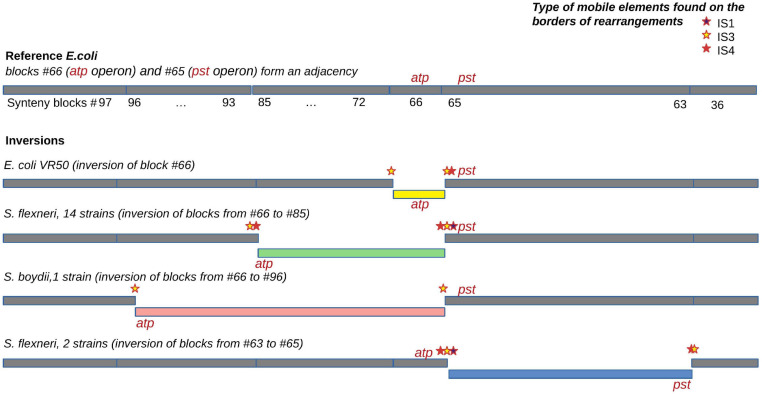
An example of an independent disruption of the adjacency between synteny blocks #65 containing the *pst* operon (high-affinity phosphate transport system) and synteny blocks #66 containing the *atp* operon (proton-translocating ATPase) by four different inversions. IS families found in the adjacency and putatively responsible for the inversions are marked by coloured stars.

For the universal synteny blocks, we found 25 parallel inversions and 40 rearrangement hotspots where a region had been involved in inversions several times independently (Supplementary Table 3). Some of these observations were easily explained by large regions between common single-copy synteny blocks that did not allow for an accurate reconstruction of the rearrangement breakpoints (Figure 5B). For other events, we detected the disruption of synteny by ISs that had been independently integrated into the same locus and then involved in different rearrangements. For instance, independent disruption of the regions between the *pst* operon (high-affinity phosphate transport system) and the *atp* operon (proton-translocating ATPase) occurred in four independent branches and participated in four different inversions (Figure 7). Focusing on regions that were strongly syntenic in *E. coli* but involved in rearrangements in *Shigella*, we found two adjacencies that had been disrupted independently in *S. sonnei*, *S. dysenteriae*, and *Shigella flexneri*. One more interesting event is the independent inversion of the Na+/H+ antiporter gene in the *S. sonnei* and *S. flexneri* lineages.

We reconstructed the history of inversions in 51 strains. This dataset included all *Shigella* spp., closely related *E. coli*, and several representative *E. coli* from each cluster without *Shigella* (Supplementary Figure 5). Of 114 reconstructed inversion events, 103 were mapped to *Shigella* branches, while only 11 corresponded to *E. coli* branches. Of these inversions, 33 were mapped to the branch separating two *S. dysenteriae*; three other *Shigella* species demonstrated rearrangements at internal and terminal branches. These results are consistent with the estimation of rearrangement rates using the number of syntenic regions as the indicator.

#### Deletions

In addition to IS accumulation, *Shigella* adaptation had been accompanied by massive pseudogenisation that in total resulted in genome reduction (Feng et al., 2011). These trends are well-known features of many pathogenic and symbiotic bacteria (Ashida et al., 2007). Indeed, *Shigella* have a significantly smaller genome size than all pathogenic and non-pathogenic *E. coli* (Supplementary Figure 6a); at that, note that the genome size of the two most abundant *E. coli* pathovars (ExPEC and STEC) is larger than that of non-pathogenic *E. coli* (Supplementary Figures 6b,c). Taking into account the high rate of non-homologous recombination, we anticipated seeing an increased rate of loss of non-universal synteny blocks in *Shigella*. We identified parallel insertions, deletions, and duplications in 2,256 out of 3,642 non-universal synteny blocks across the *E. coli/Shigella* phylogenetic tree (Supplementary Table 4). Three blocks lost in all *Shigella* and only two *E. coli* had affected the propionate catabolism (*prpABCDER*) operon. However, we did not find any strictly *Shigella-*specific large-scale deletion events.

#### Insertions

**FIGURE 8:**
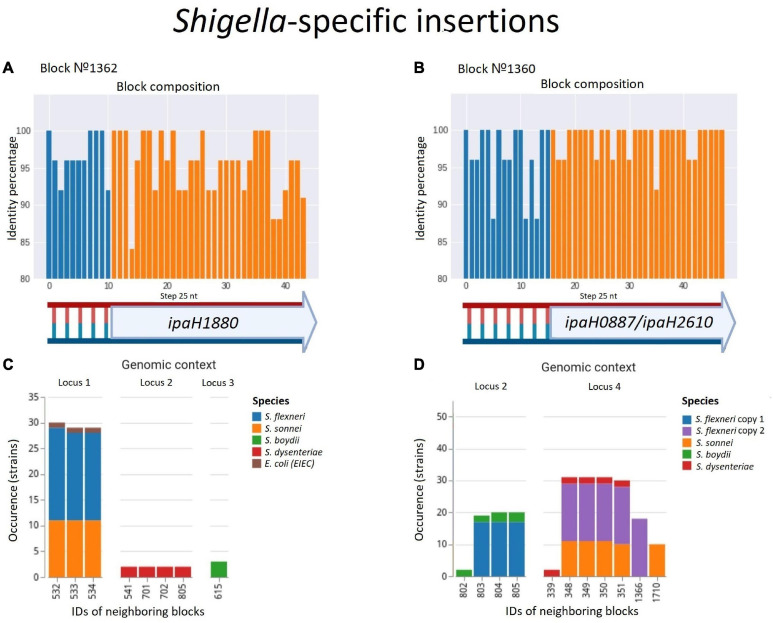
Two parallel *Shigella-specific* insertions. **(A,B)** Both inserted blocks contain long non-coding regions with more than 85% identity and the *ipaH* genes encoding E-3 ubiquitin-ligases with different substrate-specific domains. The identity was calculated for nucleotide alignments of the synteny blocks separately with step 25 nt. **(C,D)** The location of the inserted blocks in different genomic contexts confirms an independent acquisition of the block by different *Shigella* lineages; in *Shigella dysenteriae*, block #1360 has been inserted twice at different loci. We took into consideration up to five of the nearest blocks, both in the upstream and downstream direction, at the distance not exceeding 10 kb. Colours correspond to *Shigella* lineages. The gene composition of the neighbouring blocks for both insertions is shown in Supplementary Table 5.

In contrast, we found one single-copy synteny block that was present in *Shigella* and EIEC but absent in other *E. coli*, likely indicating acquisition of this block in *Shigella* rather than multiple independent losses in *E. coli*. This region contained the gene *ipaH1880* encoding an E3 ubiquitin-protein ligase, one of *Shigella* invasion-plasmid antigens, with a highly conserved 270 bp upstream non-coding region (Figure 8A). In *S. sonnei, S. flexneri*, and EIEC the fragment was integrated into the same locus, while in *S. boydii* and *S. dysenteriae*, this fragment was found in other loci (Supplementary Table 5). Although the *ipaH* genes are often surrounded by prophage genes and ISs, they do not form stable genomic islands. Thus, the mechanism of *ipaH* integration in chromosomes is uncertain.

One more block was present only in *Shigella* but absent in other *E. coli* including EIEC. This region also contained the genes *ipaH0887/ipaH2610* encoding another E3 ubiquitin-protein ligase, preceded by another conserved non-coding region of 230 nt (Figure 8B). In the chromosomes of *S. flexnerii*, we identified two copies of this block, located at a substantial distance from each other; in some strains, one of the gene copies had been annotated as a pseudogene. There are two possible evolutionary scenarios explaining this duplication. One is gene duplication in the common ancestor of *S. flexneri*; the other explanation is an independent acquisition of the copies by horizontal transfer. Based on adjacent blocks, the fragments in different strains cluster into two groups; *S. sonnei* and *S. dysenteriae* have their only copy in the first locus, while *S. boydii* has its only copy in the second locus (Supplementary Table 5). This pattern may be explained by the independent transfer of the genes via site-specific insertion or via homologous recombination, as these mechanisms retain the gene environment.

The evolutionary history of syntenic blocks seems relevant to the functional specificity of *Shigella* and EIEC, as the products of the *ipaH* genes are secreted by intracellular bacteria via the type III secretion system (T3SS) (Ashida et al., 2007) and, therefore, the *ipaH* genes repertoire may confer *Shigella-* or EIEC-specific functionality. Sequence conservation of the non-coding region indicates the importance of this sequence either for the integration or for the regulation of the *ipaH* transcription. Being highly conserved in different *Shigella* strains, these non-coding fragments are found only in the *ipaH* upstream regions and are not homologous for two different *ipaH* genes. Thus, we tentatively suggest that these non-coding fragments contain regulatory elements and play a role in the *Shigella* pathogenicity.

### Rates of Homologous Recombination

**FIGURE 9:**
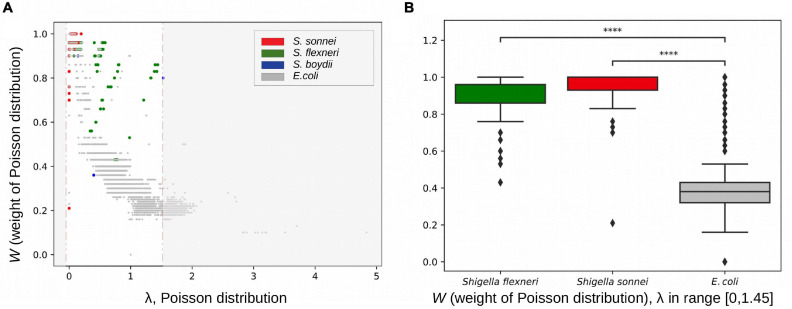
Rates of homologous recombination. **(A)** Fractions of vertically inherited fragments in strains from the B1 phylogroup calculated as the weight of the Poisson distribution (see section “Materials and Methods”). **(B)** The weight of the Poisson distribution in *Shigella* and *E. coli* in the same interval of evolutionary distances between strains. ****p < 0.0001.

We hypothesised that disruption of syntenic regions should decrease the rate of homologous recombination. To check this, we calculated fractions of horizontally transferred fragments in strains using pairwise genome alignments of *E. coli*, *S. flexneri*, *S. boydii*, and *S. sonnei* (see section “Materials and Methods”). Indeed, at the same level of divergence between strains (with the Poisson λ parameter in the vertically inherited fraction ranging from 0 to 1.45), pairs of *Shigella* strains had a significantly lower fraction of fragments horizontally transferred by homologous recombination, (1–*W*) = 0.094 ± 0.017, in comparison to pairs of *E. coli* strains, (1–*W*) = 0.612 ± 0.002 (*p* = 4.17 × 10^–124^, the Wilcoxon test) (Figure 9).

The estimated parameter λ may reflect many evolutionary parameters such as the generation time and the intensity of selection. However, the observation that a larger Poisson lambda corresponds to a lower clonal fraction of the genome, as expected, demonstrates the consistency of our results. We used this technique for *Escherichia* pairs and *Shigella* pairs at the same level of sequence similarity and hence demonstrated that the clonal genome fraction is larger in the latter, confirming our hypothesis.

## Discussion

Compared to other pathogenic *E. coli*, *Shigella* (i) accumulated a large variety of ISs, (ii) acquired new chromosomal genes, (iii) experienced exceptionally high rates of intragenomic rearrangements, and (iv) had a decreased rate of homologous recombination.

The diversity of *E. coli* pathotypes is explained by the high plasticity of its genome, as genes responsible for pathogenic traits are mostly acquired by extensive horizontal gene transfer and are often conveyed by mobile genetic elements (Touchon et al., 2009; Dobrindt et al., 2010; van Elsas et al., 2010). Both *Shigella* and EIEC spend much of their life cycle within eukaryotic cells and share many invasion-related functional systems. The adaptation to an intracellular lifestyle was conferred by the acquisition of the *pINV* plasmid encoding a T3SS (Ashida et al., 2007). Phylogenetic analysis supports the hypothesis that four existing *Shigella* lineages and the EIEC strain have arisen from different ancestral *E. coli* strains on multiple independent occasions (Pupo et al., 2000; Lan et al., 2004; Yang et al., 2007; Chaudhuri and Henderson, 2012).

The delivery of bacterial virulence proteins, called “effectors,” into host cells via T3SS plays a crucial role in the infection strategies of *Shigella*. Such effectors are involved in the reorganisation of the host cell cytoskeleton and in the modulation of cellular signalling pathways that allow the bacteria to evade the host’s immune response (Mattock and Blocker, 2017). Each IpaH family protein likely has a specific host target protein due to the substrate recognition domain, and thus makes a distinct contribution to bacterial pathogenesis (Ashida et al., 2007). Most T3SS effectors are encoded by plasmid *pINV* genes, while the biological role of chromosomally encoded *ipaH* genes remains obscure. Based on our results, we hypothesize that some chromosomal E3 ubiquitin-protein ligases are essential for the *Shigella*/EIEC pathogenicity while others may provide specific advantages. The differences in the composition of functional types of ubiquitin-protein ligases may also explain the differences in the infectious dose and disease severity between *Shigella* and EIEC pathotypes.

High numbers and the variety of mobile elements in evolutionarily young intracellular pathogens is usually explained by weaker selection against repetitive elements due to a decreased effective population size (Bordenstein and Reznikoff, 2005; Mamirova et al., 2007; Siguier et al., 2014). IS elements may drive the evolution of chromosome organisation by causing non-homologous recombination (Raeside et al., 2014). Genome rearrangements change the chromosomal architecture, which can alter gene composition and gene expression. These events are largely detrimental for free-living bacteria and are expected to be selected against (Darling et al., 2008). On the other hand, in a new environment, non-homologous recombination and the associated functional changes may provide a base for adaptation (Yan et al., 2018; Brandis and Hughes, 2020). For instance, *Burkholderia mallei*, a young obligate intracellular pathogen, has lost numerous clusters of genes through IS-mediated elimination as demonstrated by the comparison of its genome to strains of the ancestral species, *Burkholderia pseudomallei* (Losada et al., 2010; Bochkareva et al., 2018a). The genome reduction of *B. mallei* reaches 30% in some strains and the adaptation is still not complete (Mira et al., 2006). In comparison to other *E. coli*, *Shigella* spp. have slightly smaller genomes and their evolution has been accompanied by IS-mediated pseudogenisation, but not large-scale deletions.

Insertion sequence families differ in the expansion rate in *Shigella* lineages, which is expected given the independent origin of the latter. Two types of IS, IS3, and IS4, demonstrated high expansion rates in all *Shigella* lineages; this observation is in agreement with a recently published analysis of draft *Shigella* and EIEC genomes (Hawkey et al., 2020). These ISs are not common for *E. coli* populations but are a part of *pINV* that explains their expansion after the plasmid acquisition. In contrast, IS1 is present in many pathogenic *E. coli* and the difference in its frequency in pathogenic *E. coli* and *S. sonnei* is not statistically significant. The number of IS elements and, consequently, the rate of genome rearrangements in the EIEC strain were comparable with those in other *E. coli*. On the other hand, draft EIEC genomes featured larger IS frequencies in EIEC populations in comparison to other *E. coli*, but lower than in *Shigella* strains (Hawkey et al., 2020); but the rates of genomic rearrangements were not estimated in the cited paper as the studied genomes had not been assembled. Taken together, we propose that differences in the number of IS genomic elements may have influenced different stages of formation of intracellular pathogens EIEC and *Shigella* spp.

An expected consequence of frequent genome rearrangement is a decrease in the rate of homologous recombination. Indeed, in comparison to *E. coli*, *Shigella* genomes contain fewer DNA segments horizontally transferred by homologous recombination. However, this also could be explained by a smaller population size and an isolated intracellular lifestyle of *Shigella* strains. The levels of homologous recombination in core genes, manifesting as an incongruence of gene phylogenetic trees with the strain phylogeny, are relatively smaller in endosymbionts and intracellular pathogens (González-Torres et al., 2019). These are likely interconnected processes. Bottlenecks and decreased selection pressure lead to the increase in the number of IS elements (Mira et al., 2006; Mamirova et al., 2007); this in turn provides more opportunities for genome rearrangements that become tolerated due to decreased selection. Indeed, bursts of rearrangements were observed in the genomes of pathogens that had recently changed the host and lifestyle, such as *Yersinia pestis* (Bochkareva et al., 2018a) and *B. mallei* (Bochkareva et al., 2018b). On the other hand, the relative isolation of strains with a mainly intracellular lifestyle provides fewer opportunities for homologous recombination, while the lack of genome collinearity creates mechanistic obstacles to the process (Aguilera and Rothstein, 2007). At that, neither increased rearrangement rate nor decreased homologous recombination rate is observed in intracellular EIEC strains of *E. coli*, supporting the link between these phenomena.

The analysis of genome rearrangements requires complete genomes, while less than 1% of available *Shigella* genomes have a sufficient quality of assembly. Another issue is that misassemblies are mainly caused by genomic repeats and may be confused with true rearrangements. Although there are several strategies widely used for assembly validation such as long read (re)sequencing and/or PCR contiguity verification (English et al., 2012; Madoui et al., 2015; Acuña-Amador et al., 2018), some of the detected rearrangements may have been caused by inaccuracies in gap closure procedures. While each particular observation requires experimental verification, the observed correlation between rearrangement rates and mutation rates in closely related strains allow us to conclude that assembly errors do not affect the evolutionary signal in the analysed data (Bochkareva et al., 2018b).

Due to high genomic plasticity, pathogenic *E. coli* are among the most frequent causes of bacterial infections in humans (Allocati et al., 2013; Pasqua et al., 2017). In particular, the *E. coli* O104:H4 outbreak in Germany in 2011 was caused by a strain that had acquired characteristics of two previously described pathotypes (Burger, 2012). The emergence of antibiotic-resistant *Shigella* with the absence of an effective vaccine highlights the importance of detailed studies of this pathogen (Hassan et al., 2020). Our results contribute to the understanding of genomic properties associated with adàptation to the intracellular lifestyle of *Shigella* and EIEC and the developed approaches should be broadly applicable to other young bacterial pathogens.

## Data Availability Statement

The datasets presented in this study can be found in online repositories and downloaded via the link https://github.com/zseferbekova/ShigellaProject. The names of the repository/repositories and accession number(s) can be found in the article/Supplementary Material.

## Author Contributions

MG and OB conceived and designed the study. ZS, RA, AZ, and NA developed the methods. ZS, YY, RA, ND, and OB analysed the data. ZS, NA, OB, and MG wrote the manuscript. All authors read and approved the final version of the manuscript.

## Conflict of Interest

The authors declare that the research was conducted in the absence of any commercial or financial relationships that could be construed as a potential conflict of interest.

## Supplementary Material

Supplementary Figure 1 Phylogenetic trees. **(a)** *Escherichia coli* phylogenetic tree. The tree is based on the nucleotide alignment of universal single-copy orthologs. Strains shown in red were excluded from further analysis. **(b)** *Escherichia coli* and *Shigella* phylogenetic tree. The tree is based on the nucleotide alignment of universal single-copy orthologs with a 95% identity threshold.

Supplementary Figure 2 Construction of breakpoint graph. **(a)** Genome graphs of unichromosomal circular genomes *P* = (0, 1, 2, 3, 4, and 5) and *Q* = (0, 1, −4, −3, −2, and 5), the adjacency edges of the genome *P* (left) are shown in blue, the edges of the genome *G* (right) are shown in red. **(b)** The breakpoint graph *G*(*P, Q*) of genomes *P* and *Q*. **(c)** The multiple breakpoint graph of five unichromosomal circular genomes.

Supplementary Figure 3 Chromosome maps. **(a)** *Escherichia coli* C4, **(b)** *Escherichia coli* cfsan029787, **(c)** *Shigella flexneri* 1a 228, **(d)** *Shigella boydii* atcc 9210, **(e)** *Shigella dysenteriae* sd197, and **(f)** *Shigella sonnei* fdaargos 524. The inner circle – GC-skew, the second (blue) circle – synteny blocks, the third (green) circle – universal synteny blocks, the fourth (grey) circle – ISs, and the fifth (grey) circle – density of ISs.

Supplementary Figure 4 Phylogenetic distance versus the number of synteny blocks for each pair of genomes. **(a)** In phylogroup A, **(b)** in phylogroup B2, **(c)** in phylogroup D, **(d)** in phylogroup F. Each point represents a pair of genomes and is coloured according to the genomes the pair includes.

Supplementary Figure 5 Inversion events reconstructed by the MGRA software. **(a)** A cladogram with the numbers of inversions shown for each branch. **(b)** The corresponding phylogenetic tree. Phylogroups are marked with coloured strips, pathogenic strains are shown in different colours. Both trees are unrooted. The trees are also available online (iTOL): https://itol.embl.de/tree/9318063252480721583313649.

Supplementary Figure 6 Genome sizes in panel **(a)** *Shigella*, non-pathogenic *E. coli*, other pathogenic *E. coli*, and all *E. coli* (excluding *Shigella* spp.). **(b)** Two most abundant pathovars – ExPEC and STEC, non-pathogenic *E. coli* and all *E. coli* (excluding *Shigella* spp.). **(c)** Different groups of *E. coli*. The numbers above boxplots indicate the number of genomes in each group. The Wilcoxon–Mann–Whitney test with the Bonferroni correction: ^∗^*p* ≤ 0.05; ^∗∗^*p* ≤ 0.01; ^∗∗∗^*p* ≤ 0.001; ^****^*p* ≤ 0.0001.

Supplementary Table 1 The list of *Escherichia coli* and *Shigella* genomes included in the analyses.

Supplementary Table 2 Coordinates of synteny blocks.

Supplementary Table 3 Adjacencies of common synteny blocks.

Supplementary Table 4 Copy number of non-common synteny blocks.

Supplementary Table 5 Gene composition of synteny blocks surrounding *Shigella*-specific insertions.
