# Supplementary figures and images for: High Rates of Genome Rearrangements and Pathogenicity of Shigella spp
Source: Front Microbiol. 2021 Apr 12;12:628622. doi: 10.3389/fmicb.2021.628622 (PMC8072062; doi:10.3389/fmicb.2021.628622)

Tree scale: 0.01

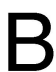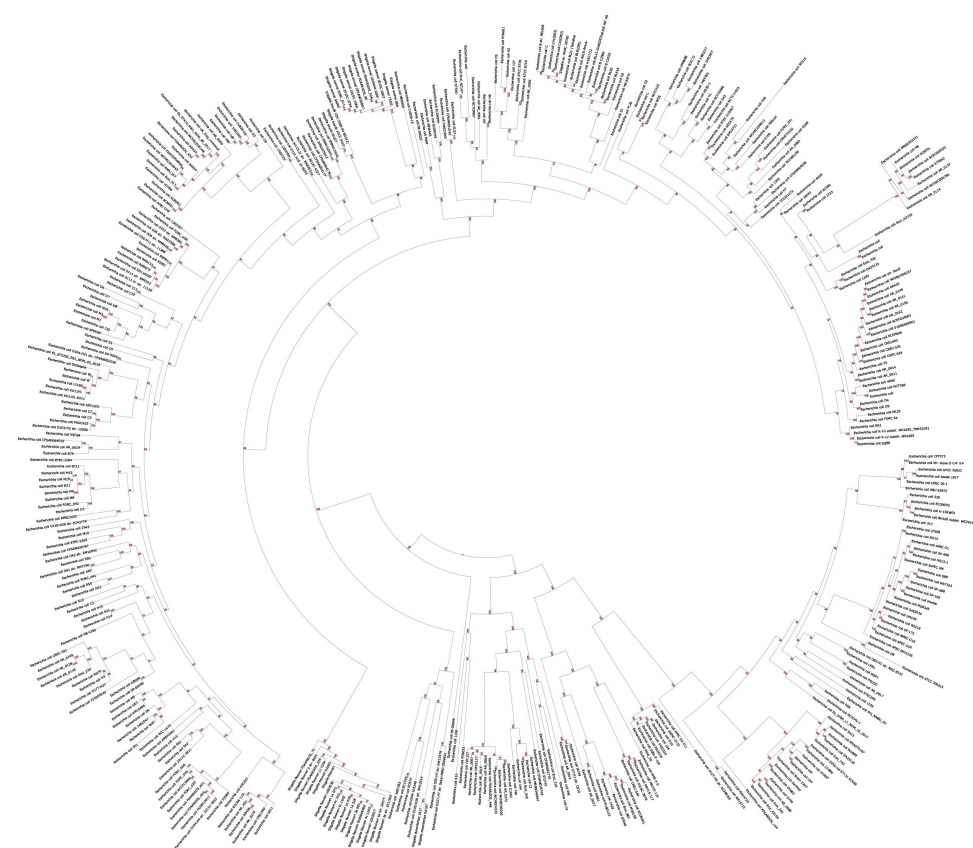

Supplement: Supplementary Figure 1 — Phylogenetic trees. (a) Escherichia coli phylogenetic tree. The tree is based on the nucleotide alignment of universal single-copy orthologs. Strains shown in red were excluded from further analysis. (b) Escherichia coli and Shigella phylogenetic tree. The tree is based on the nucleotide alignment of universal single-copy orthologs with a 95% identity threshold. [file Image_1.pdf]

(a)

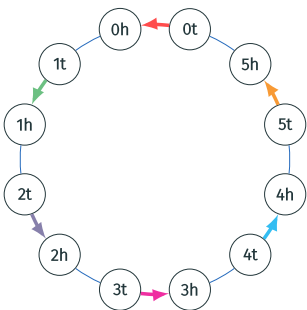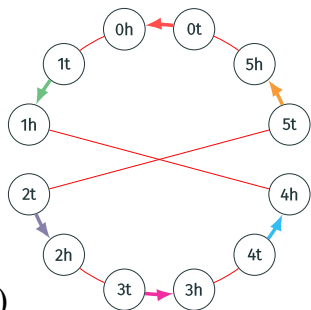

(b)

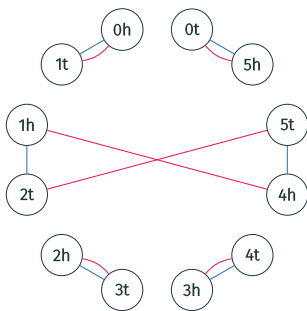

(c)

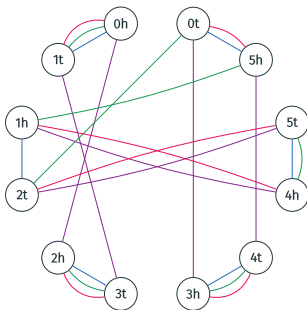

Supplement: Supplementary Figure 2 — Construction of breakpoint graph. (a) Genome graphs of unichromosomal circular genomes P = (0, 1, 2, 3, 4, and 5) and Q = (0, 1, −4, −3, −2, and 5), the adjacency edges of the genome P (left) are shown in blue, the edges of the genome G (right) are shown in red. (b) The breakpoint graph G(P, Q) of genomes P and Q. (c) The multiple breakpoint graph of five unichromosomal circular genomes. [file Image_2.pdf]

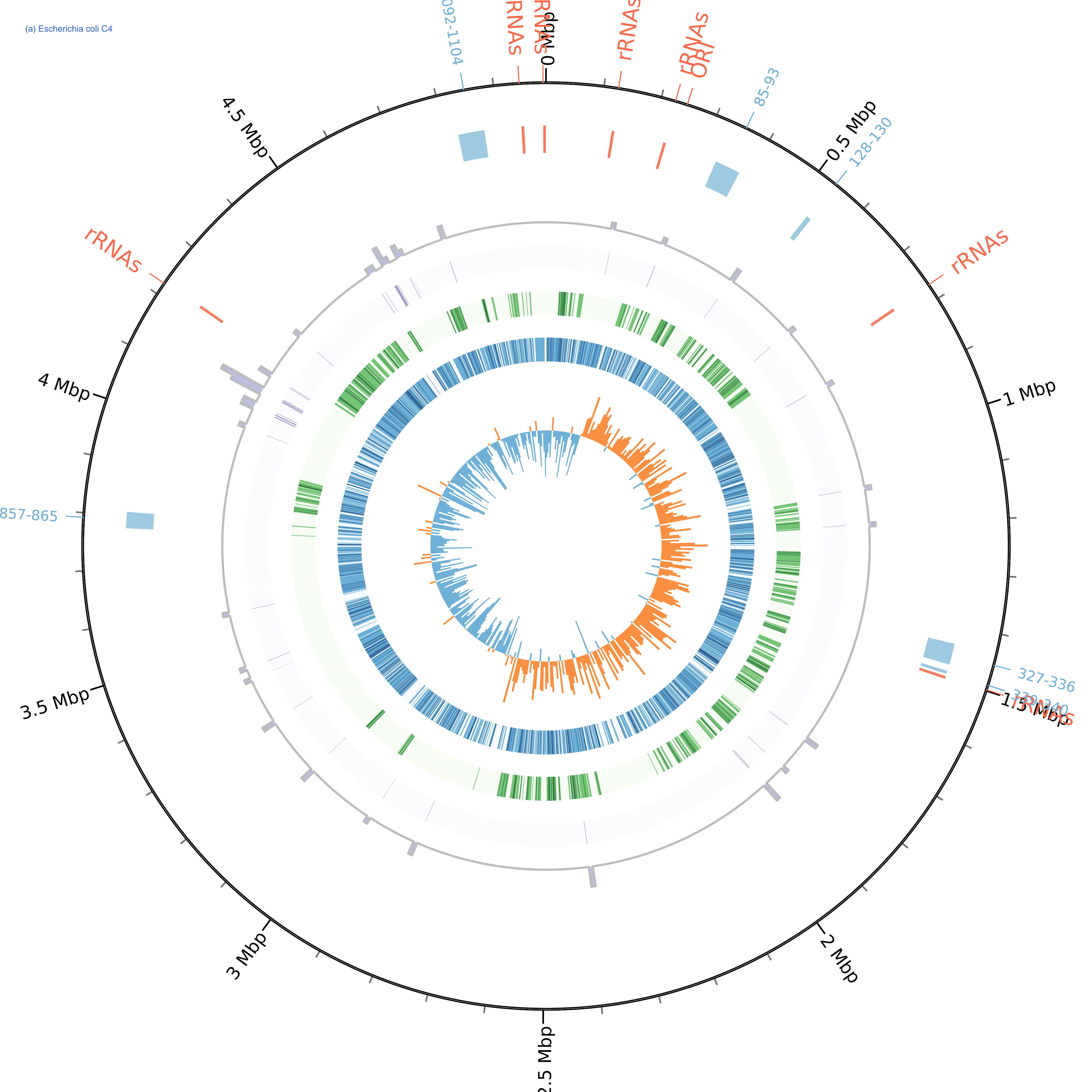

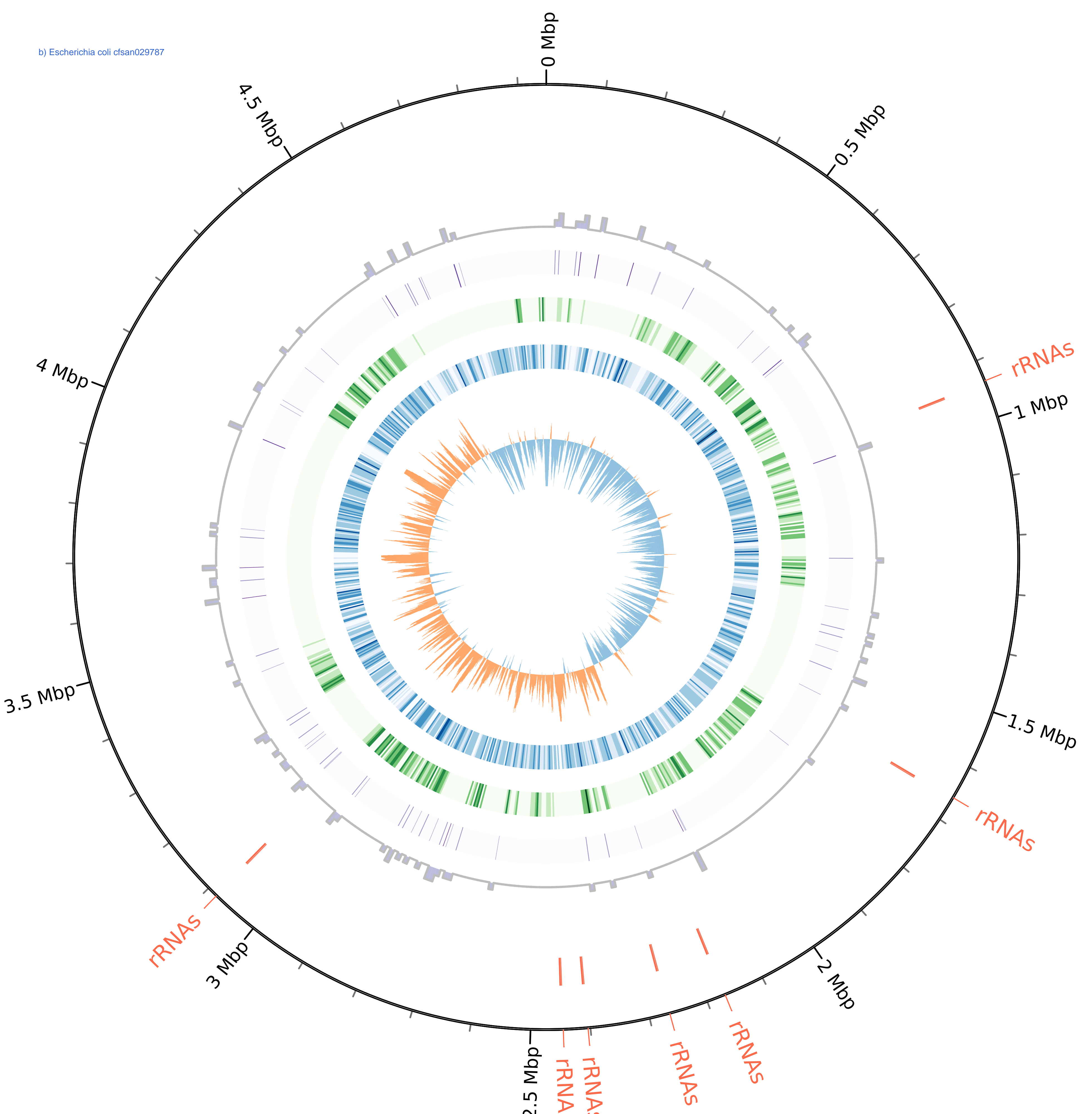

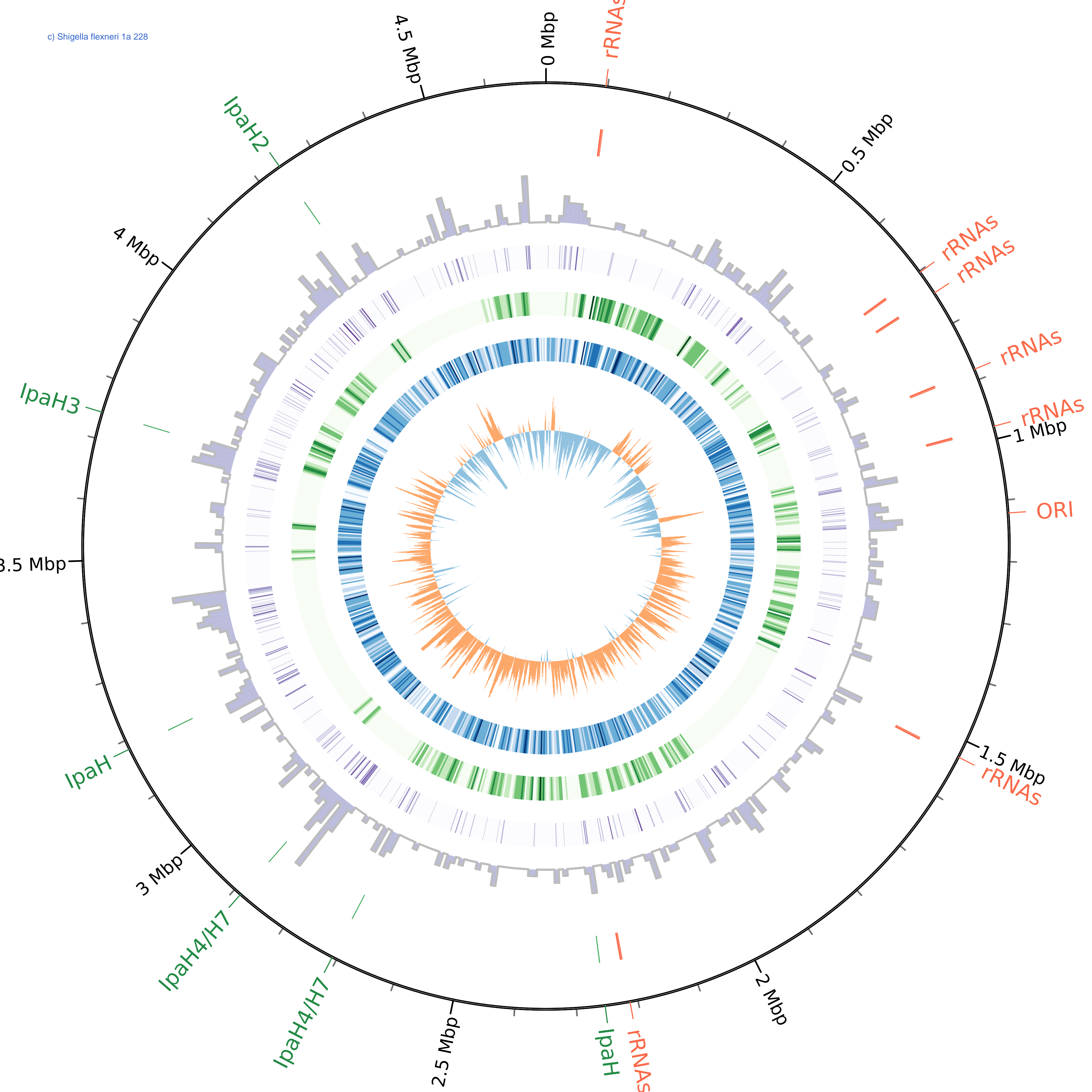

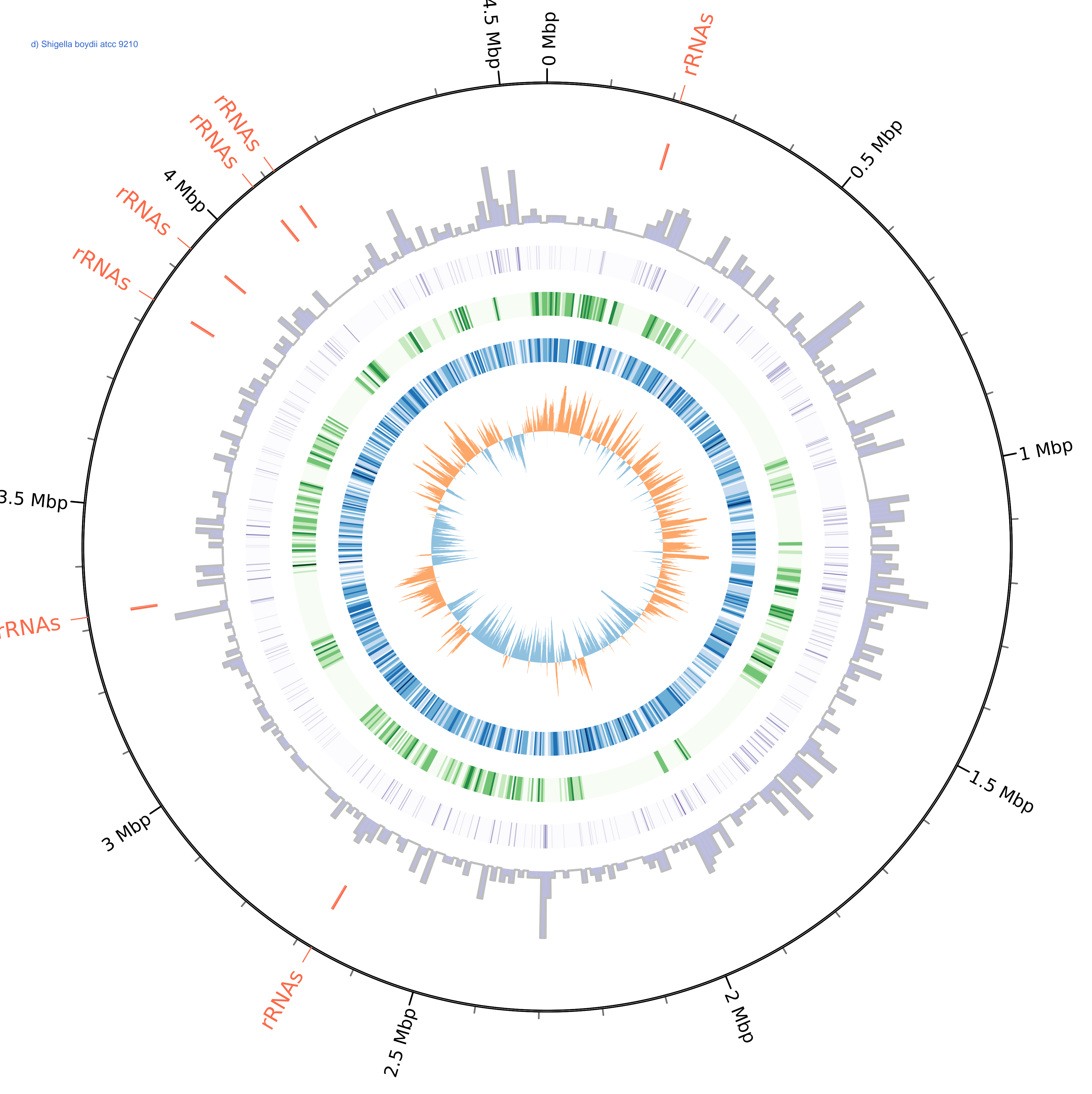

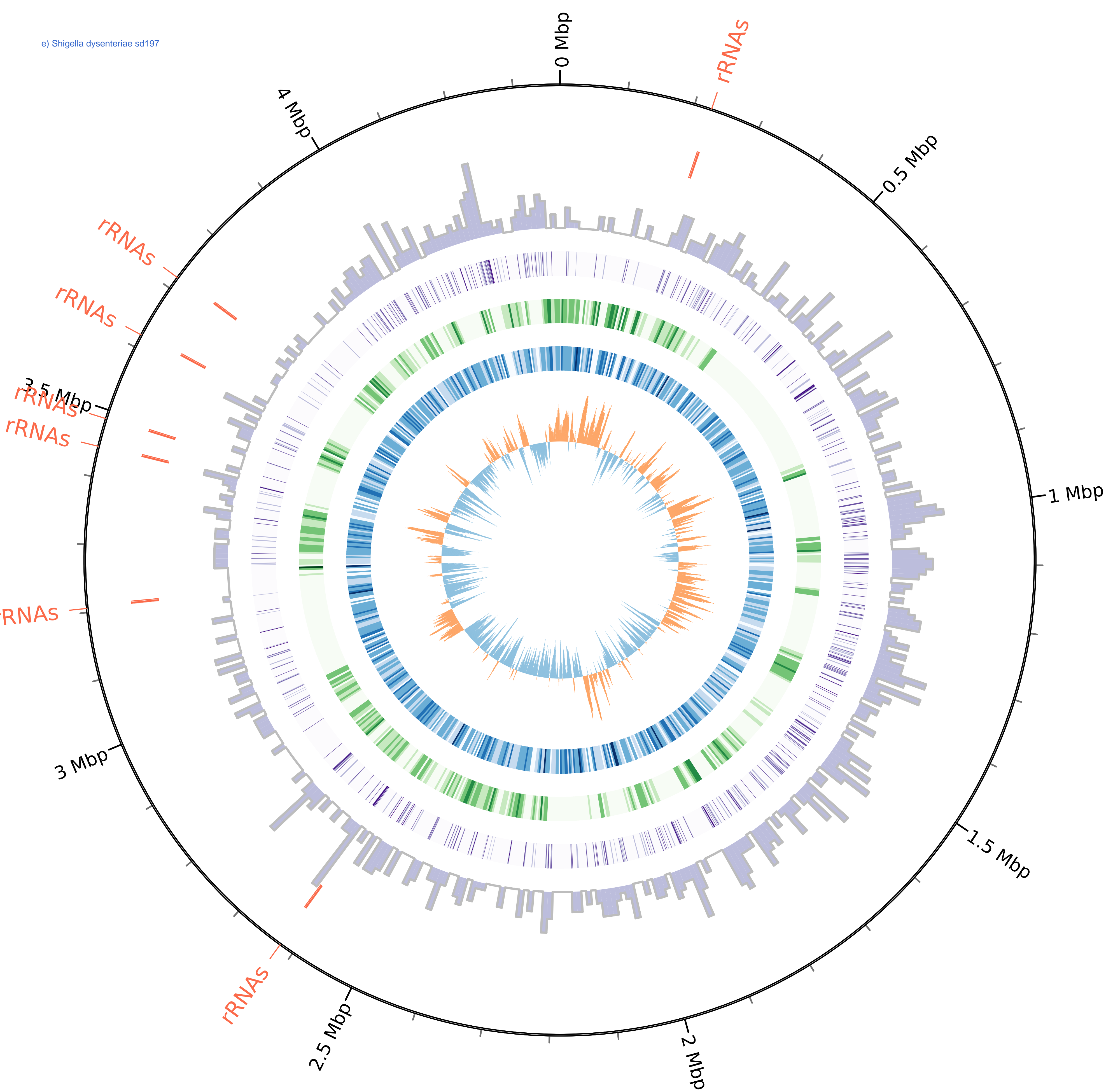

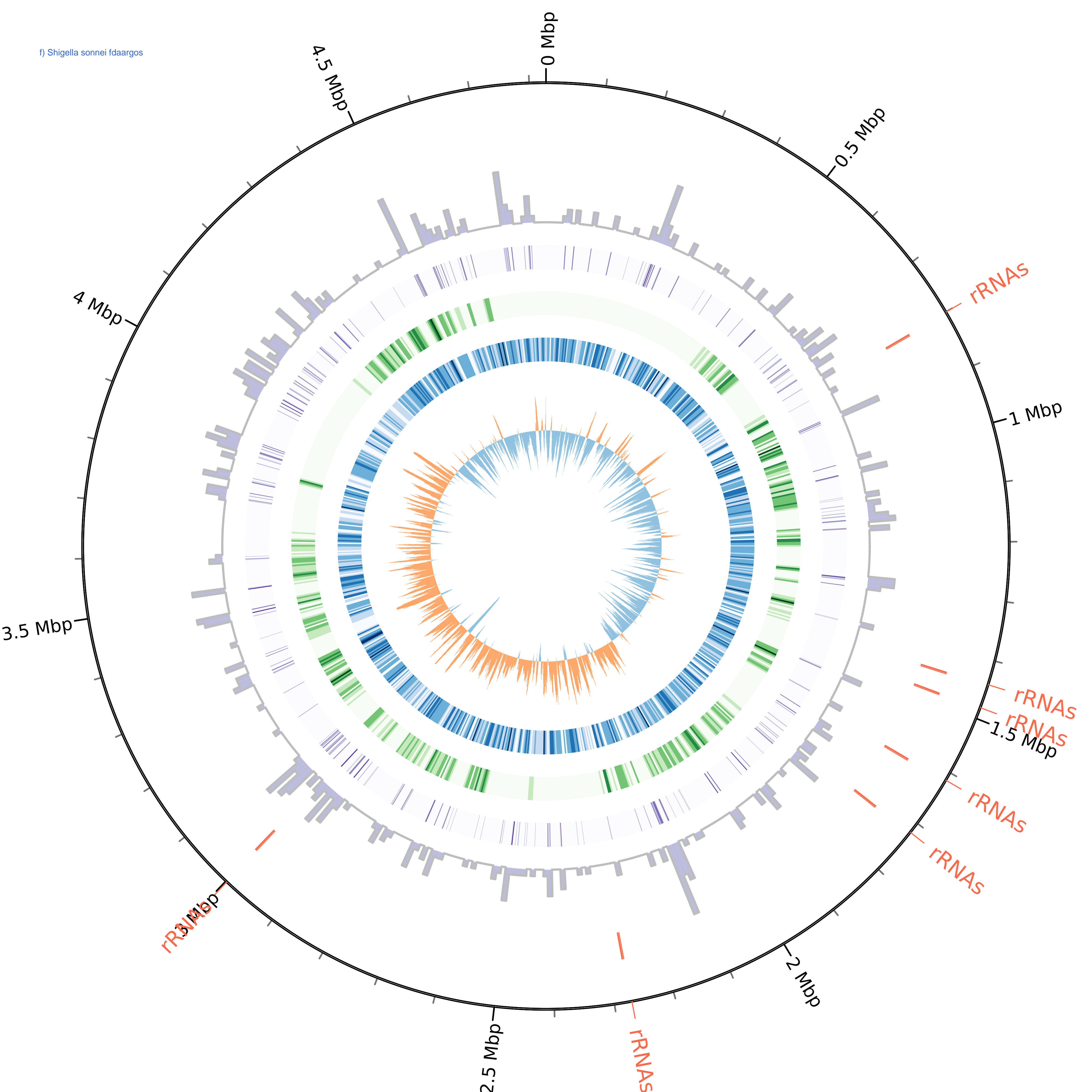

Supplement: Supplementary Figure 3 — Chromosome maps. (a) Escherichia coli C4, (b) Escherichia coli cfsan029787, (c) Shigella flexneri 1a 228, (d) Shigella boydii atcc 9210, (e) Shigella dysenteriae sd197, and (f) Shigella sonnei fdaargos 524. The inner circle – GC-skew, the second (blue) circle – synteny blocks, the third (green) circle – universal synteny blocks, the fourth (grey) circle – ISs, and the fifth (grey) circle – density of ISs. [file Image_3.pdf]

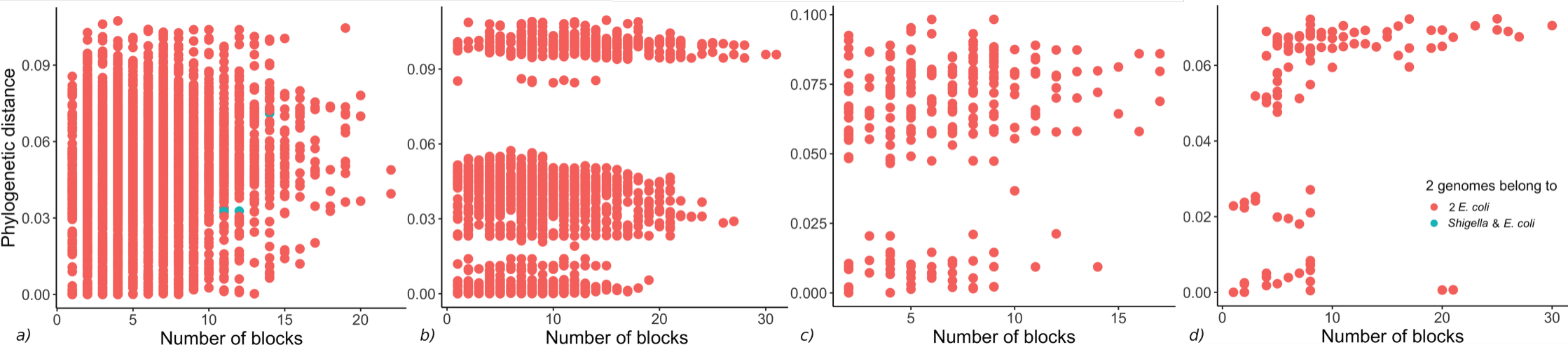

Supplement: Supplementary Figure 4 — Phylogenetic distance versus the number of synteny blocks for each pair of genomes. (a) In phylogroup A, (b) in phylogroup B2, (c) in phylogroup D, (d) in phylogroup F. Each point represents a pair of genomes and is coloured according to the genomes the pair includes. [file Image_4.pdf]

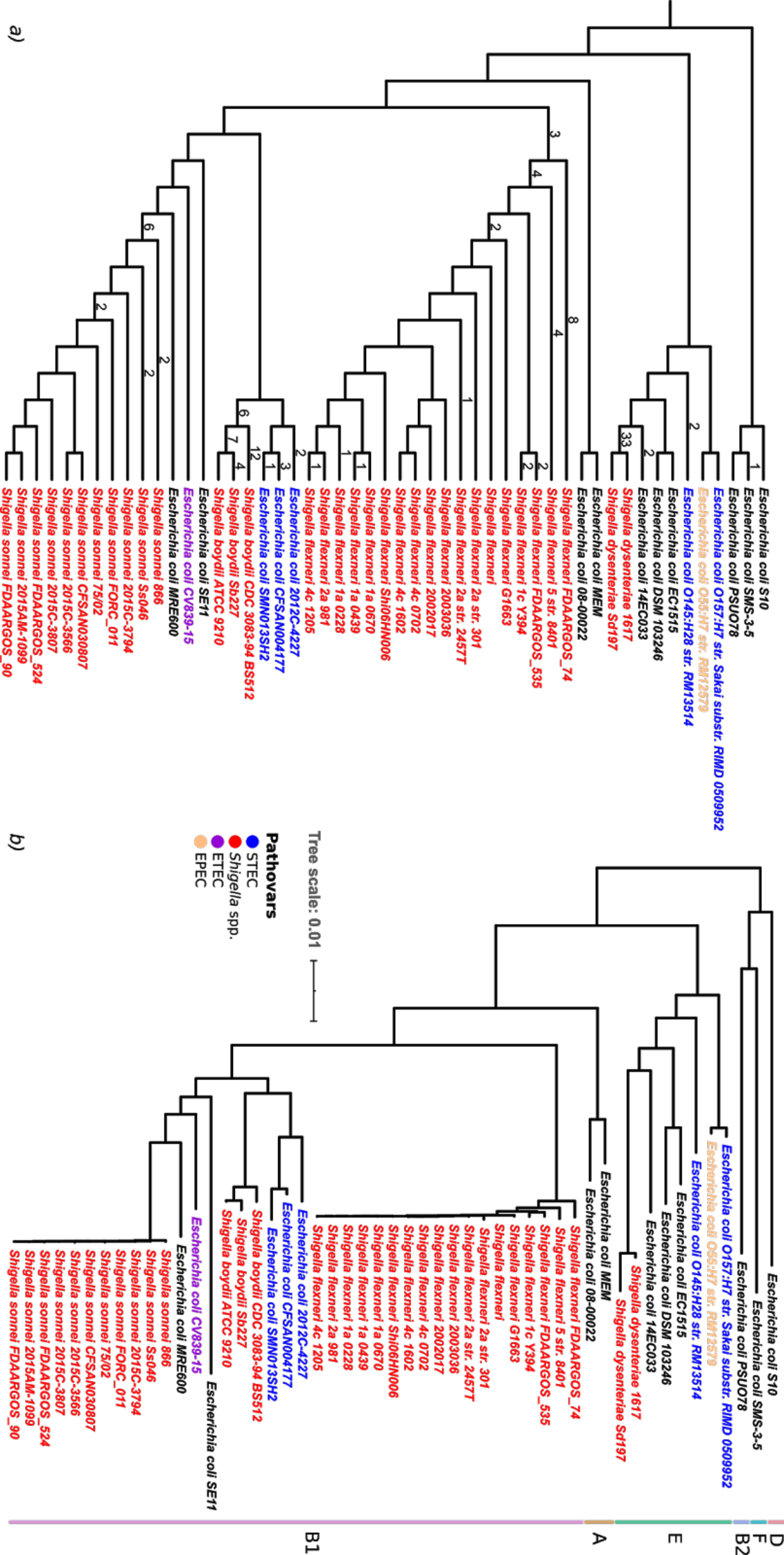

Supplement: Supplementary Figure 5 — Inversion events reconstructed by the MGRA software. (a) A cladogram with the numbers of inversions shown for each branch. (b) The corresponding phylogenetic tree. Phylogroups are marked with coloured strips, pathogenic strains are shown in different colours. Both trees are unrooted. The trees are also available online (iTOL): https://itol.embl.de/tree/9318063252480721583313649. [file Image_5.pdf]

(a)

(b)

(c)

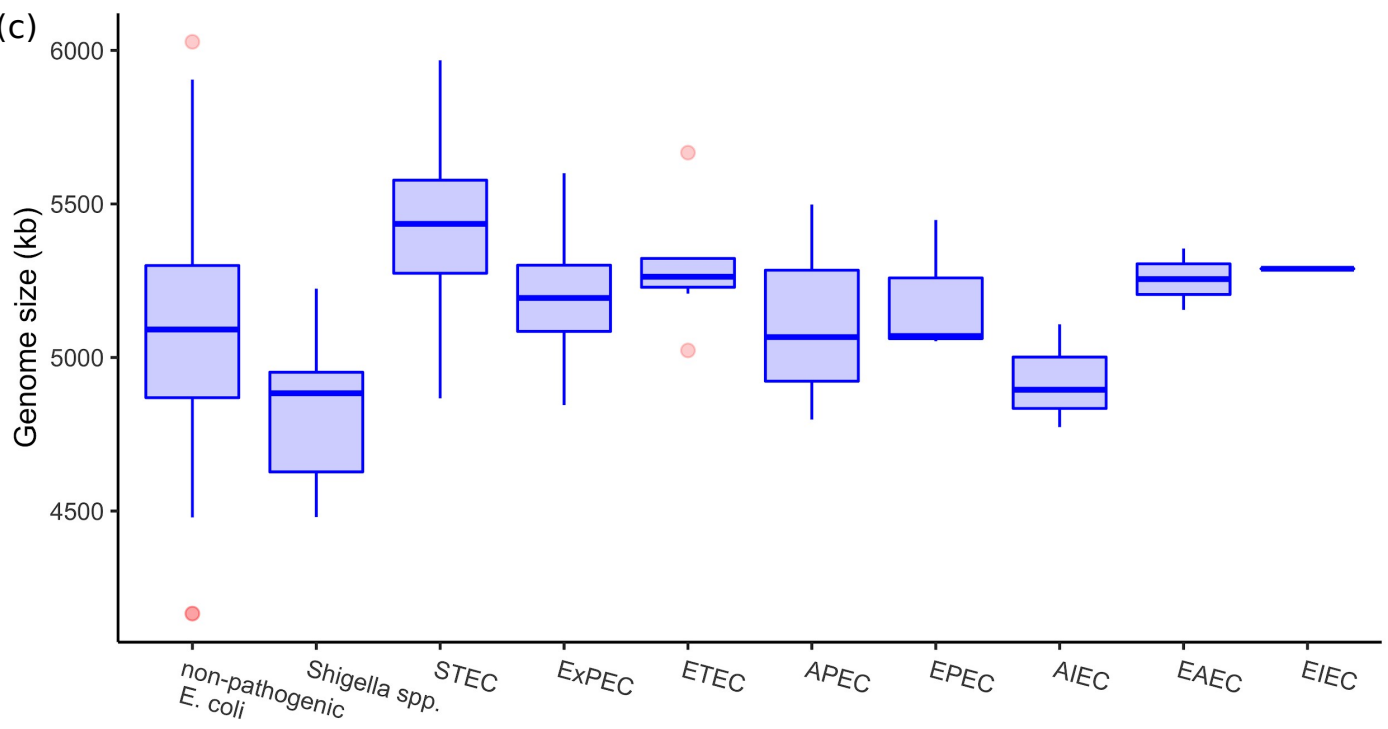

Supplement: Supplementary file 7 [file Image_7.PDF]
