# Supplementary material for: High Rates of Genome Rearrangements and Pathogenicity of Shigella spp
Source: Front Microbiol. 2021 Apr 12;12:628622. doi: 10.3389/fmicb.2021.628622 (PMC8072062; doi:10.3389/fmicb.2021.628622)
Supplement: Supplementary Figure 6 — Genome sizes in panel (a) Shigella, non-pathogenic E. coli, other pathogenic E. coli, and all E. coli (excluding Shigella spp.). (b) Two most abundant pathovars – ExPEC and STEC, non-pathogenic E. coli and all E. coli (excluding Shigella spp.). (c) Different groups of E. coli. The numbers above boxplots indicate the number of genomes in each group. The Wilcoxon–Mann–Whitney test with the Bonferroni correction: ∗p ≤ 0.05; ∗∗p ≤ 0.01; ∗∗∗p ≤ 0.001; ****p ≤ 0.0001. [file Image_6.pdf]

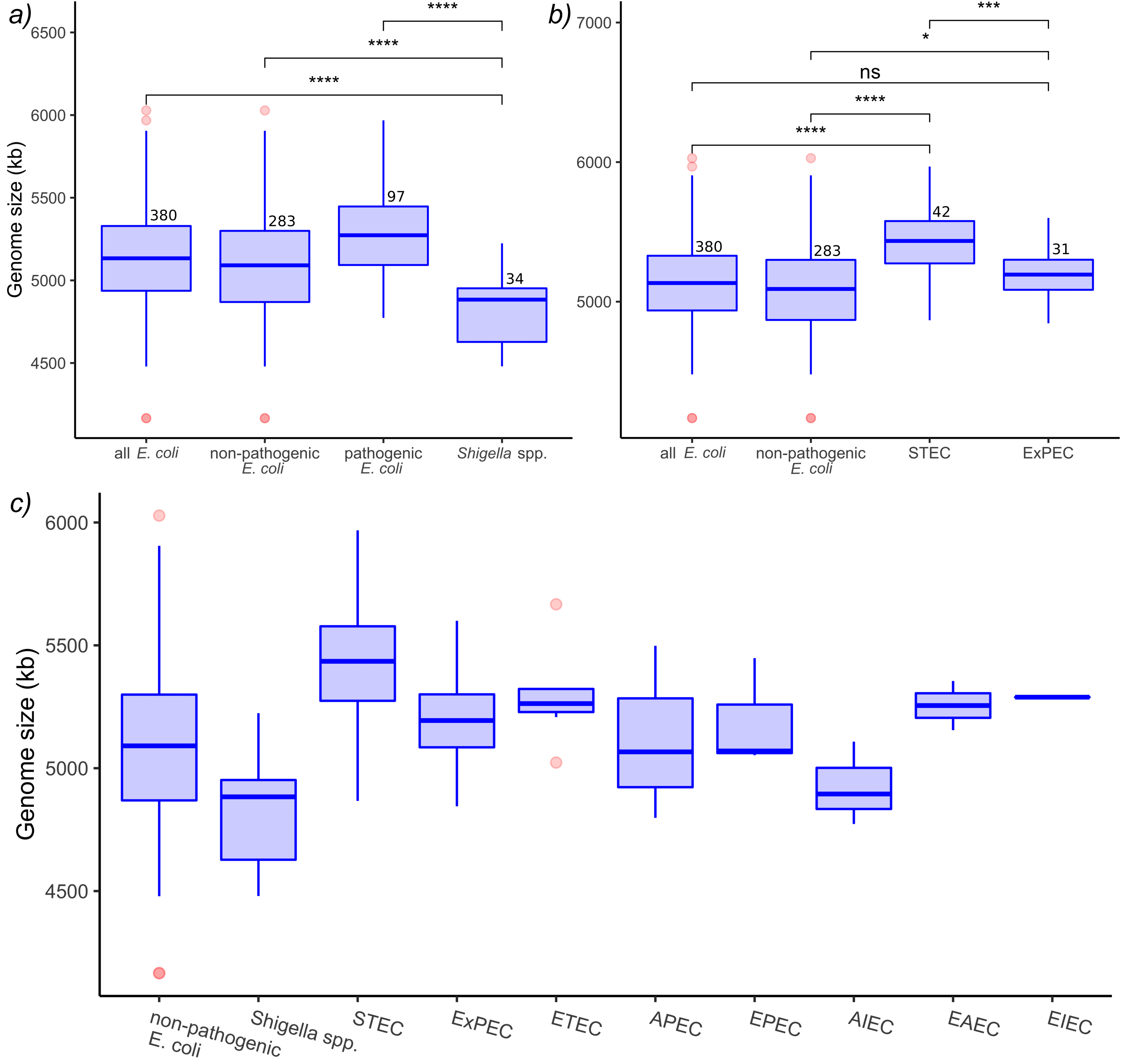
a)
Genome size (kb)
6500
6000
5500
5000
4500
****
****
****
380
283
97
34
all E. coli
non-pathogenic E. coli
pathogenic E. coli
Shigella spp.
b)
7000
6000
5000
***
*
ns
****
****
380
283
42
31
all E. coli
non-pathogenic E. coli
STEC
ExPEC
c)
Genome size (kb)
6000
5500
5000
4500
non-pathogenic E. coli
Shigella spp.
STEC
ExPEC
ETEC
APEC
EPEC
AIEC
EAEC
EIEC
